# Initial stage growth of Ge_*x*_Si_1−*x*_ layers and Ge quantum dot formation on Ge_*x*_Si_1−*x*_ surface by MBE

**DOI:** 10.1186/1556-276X-7-561

**Published:** 2012-10-09

**Authors:** Aleksandr I Nikiforov, Vyacheslav A Timofeev, Serge A Teys, Anton K Gutakovsky, Oleg P Pchelyakov

**Affiliations:** 1 Rzhanov Institute of Semiconductor Physics, Siberian Branch of the Russian Academy of Science, Lavrentjeva 13, Novosibirsk, 630090, Russia

**Keywords:** Silicon, Germany, Epitaxy, RHEED, Growth mode, Quantum dots

## Abstract

Critical thicknesses of two-dimensional to three-dimensional growth in Ge_*x*_Si_1−*x*_ layers were measured as a function of composition for different growth temperatures. In addition to the (2 × 1) superstructure for a Ge film grown on Si(100), the Ge_*x*_Si_1−*x*_ layers are characterized by the formation of (2 × *n*) reconstruction. We measured *n* for all layers of Ge/Ge_*x*_Si_1−*x*_/Ge heterosystem using our software with respect to the video recording of reflection high-energy electron diffraction (RHEED) pattern during growth. The *n* reaches a minimum value of about 8 for clear Ge layer, whereas for Ge_*x*_Si_1−*x*_ films, *n* is increased from 8 to 14. The presence of a thin strained film of the Ge_*x*_Si_1−*x*_ caused not only the changes in critical thicknesses of the transitions, but also affected the properties of the germanium nanocluster array for the top Ge layer. Based on the RHEED data, the hut-like island form, which has not been previously observed by us between the hut and dome islands, has been detected. Data on the growth of Ge/Ge_*x*_Si_1−*x*_/Ge heterostructures with the uniform array of islands in the second layer of the Ge film have been received.

## Background

The Ge/Ge_*x*_Si_1−*x*_/Ge heterosystems with alternating layers of quantum dots and quantum wells are of great practical interest for the fabrication of mid-infrared photodetectors based on intraband transitions [1]. The energy diagram is modified due to changes both in the composition and in the thickness of the Ge_*x*_Si_1−*x*_ film, as well as in the growth temperature. Potentialities of *engineering* the quantum dot electron structure are expanded when quantum dots are arranged in close proximity to the two-dimensional (2D) potential well. Such a potential well is a thin continuous layer of Ge_*x*_Si_1−*x*_ solid solution; variations in the composition and thickness of the layer allow the energy structure of the system ‘quantum dot-solid solution layer’ to be controlled [2]. There arise additional intraband transitions of charge carriers from quantum dot levels to 2D sub-bands of the solid solution in the system. In such heterostructures, optical transitions between the bound states in a quantum dot and delocalized states in the plane of the 2D sub-bands of the solid solution film have been realized. By varying the width and the germanium content in the quantum well, the necessary energy of the optical transition can be achieved. Since the initial state of the hole is a bound state in a quantum dot thereby removing the ban on the optical transitions at normal incidence of the light, thus, it is possible to produce the mid-IR photodetectors operating at normal light incidence with high detectivity [3].

Numerous discussions on regularities of the formation of Ge and GeSi islands on the Si(100) surface are available in the literature. The system under study is considered as a model for understanding the processes of island formation and evolution during the Stranski-Krastanov growth mode [4]. A specific feature of the Ge growth on the Si(100) surface is the existence of two types of islands: these are the so-called *hut* clusters and three-dimensional (3D) islands facetted by {113} planes. *Hut* clusters are faceted by {105} planes; they are dozens of nanometers in size depending on the growth conditions. Three-dimensional islands are formed on depositing Ge in a larger quantity, and they are larger in size. Theoretical calculations on critical thicknesses of the morphological transitions are reported in [5-7] and experimental data in [8-10]; numerous data were summarized in review papers [11-13]. However, only scarce data are available in the literature concerning the studies of Ge/SiGe/Si(100) structures. The surface morphology of the 15-nm strained buffer layer of Si_0.7_Ge_0.3_ solid solution was studied using the atomic force microscopy technique [14] before and after growth of Ge islands. After six monolayers of Ge has been deposited at 600°C, islands of two types are formed: small *hut* islands (edge length 20 to 45 nm, height *ca*. 2.1 nm) faceted by {105} planes and large *dome* islands (diameter *ca*. 50 nm, height *ca*. 8.7 nm). Densities of the *hut* and *dome* islands are 1 × 10^11^ and 1 × 10^9^ cm^−2^, respectively. The critical thickness of the Ge film at transition to the 3D growth mechanism was studied [15] depending on the composition of the GeSi solid solution at high deposition temperature (700°C). The islands were shown [16] to increase both in size and in density as the Ge content was increased in the pre-deposited Si_1−*x*_Ge_*x*_ solid solution. The authors of a study [16] assume that the increased island density is caused by an increased surface roughness after the SiGe deposition, while the islands increased in size due to increasing Si content that resulted from mixing at high growth temperature and from a decrease of the wetting layer in thickness. The latter is accounted for by the accumulation of elastic strain energy in the SiGe layer. Ge islands grown on the buffer layer of the solid GeSi solution produced a more intense photoluminescence signal at room temperature than the signal of Ge islands grown on Si(100). This result was obtained due to a higher quantum dot density which provides more effective capturing of charge carriers.

The effect on the surface morphology produced by the epitaxial growth of Ge on Si(001) is a rapid change of the surface reconstruction. In addition to the (2 × 1) superstructure for a Ge film grown on Si(001), the quasi-equilibrium is characterized by the formation of (2 × *n*) reconstruction [17]. The (2 × *n*) reconstruction begins to appear to release the accumulated misfit strain [18]. Since strain increases with increasing film thickness, other modes of strain relaxation become significant. The 2D to 3D transition starts to be observed at the Ge thickness greater than or equal to three monolayers.

## Methods

A molecular beam epitaxy (MBE) installation Katun-C equipped with two electron beam evaporators for Si and Ge was used for synthesis; dopants (Sb and B) were evaporated from the usual and high temperature effusion cells. Analytical equipment of the chamber included a mass spectrometer, a quartz thickness monitor, and a high-energy electron (20 kV) diffractometer. Diffraction patterns were monitored during the growth using a CCD camera that is online with a PC. The software allowed us to monitor both the whole images and chosen fragments of the diffraction patterns at the rate of ten frames per second. Ge and GeSi layers grew at the rate of ten monolayers per minute. The Ge and Si growth rates were controlled using quartz thickness monitors. Silicon 4-in diameter (100) p-type plates misoriented by less than 0.5° were used as substrates.

After chemical pretreatment, substrates were mounted in a growth chamber where they were cleaned in a low silicon flow at 800°C for 5 min. The cleaning process was controlled using reflection high-energy electron diffraction (RHEED) patterns where the appearance of proper Si(100)-(2 × 1) superstructure was identified.

RHEED was the main method used for surface analysis, and it is the most practiced technique in MBE. This technique enabled oscillations of the in-plane lattice constant to be detected for the Ge film growing according to the 2D mechanism on the silicon surface [19]. *Ex situ* scanning tunnel microscopy (STM) with an ultrahigh vacuum instrument Omicron-Riber (Omicron Nanotechnology GmbH, Taunusstein, Germany; Riber, Paris, France) was used for the characterization of the surface morphology.

## Results and discussion

**Figure 1:**
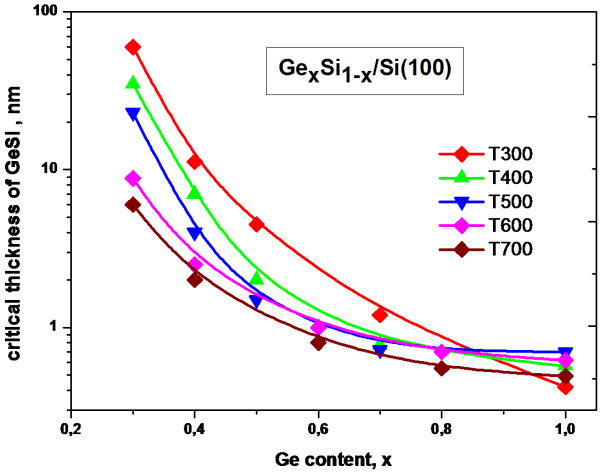
**2D to 3D transition thickness of Ge**_***x***_**Si**_**1−*x***_** layers for different Ge content and growth temperature.**

As the deposited layer increases in thickness, elastic strains induced by mismatching of the Si and Ge lattice constants also increase. Starting with some critical thickness, transitions from 2D to 3D growth mechanism are observed, with some strains being relaxed, which is energetically favorable due to a decrease in the free energy of the system. Thus, identifying the moments of 2D to 3D transitions at various thicknesses of the Ge_*x*_Si_1−*x*_ layer allowed the 2D to 3D transition thickness of the Ge film to be determined as a function of the Ge_*x*_Si_1−*x*_ thickness for different Ge content in Ge_*x*_Si_1−*x*_ layers and growth temperature (see Figure 1). The obtained dependence differs from the calculated data [5,6]. The reason is that the said experimental dependence indicates the thicknesses of the strained pseudomorphous solid solution when the 3D islands emerge, while the calculated dependences [5,6] relate to the solid solution thickness when the plastic relaxation occurs and mismatch dislocation are introduced into the interface.

**Figure 2:**
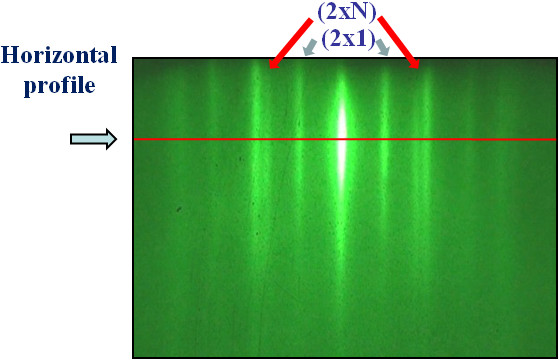
**The horizontal profile of the RHEED pattern for Ge**_***x***_**Si**_**1−*x***_** layer (*x*= 0.2).**

**Figure 3:**
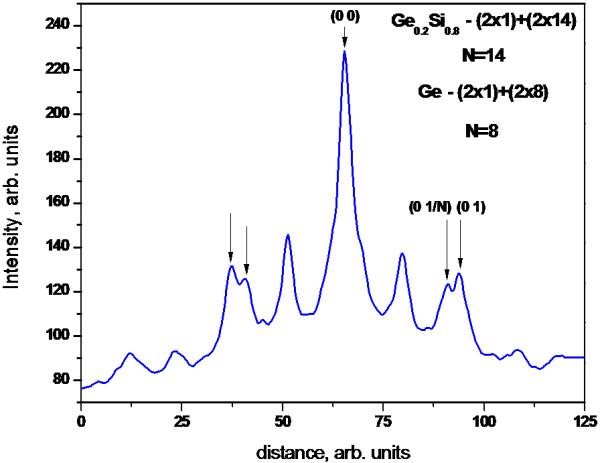
Horizontal intensity profile of the RHEED pattern.

The surface morphology of the germanium island film on the surface of Ge_*x*_Si_1−*x*_ solid solution changes essentially if germanium islands are formed as *hut* clusters before growing the Ge_*x*_Si_1−*x*_ layer. The morphology of the Ge_*x*_Si_1−*x*_ layer located above the *hut* islands depends on the Ge content in the Ge_*x*_Si_1*−*x_ layer. The Ge_*x*_Si_1−*x*_ film is a combination of (2 × 1) and (2 × *n*) reconstructions for *x* < 0.25. On the Ge_*x*_Si_1−*x*_ surface with concentrations *x* > 0.25, both the above-mentioned reconstructions and the relief of underlying islands are observed. We measured *n* of (2 × *n*) reconstruction using our software with respect to the video recording of the RHEED pattern during growth (Figure 2). The *n* reaches a minimum value of about 8 for clear Ge layer, whereas for Ge_*x*_Si_1−*x*_ films, *n* increases from 8 to 14 (Figure 3). The further deposition of the Ge film on the solid solution layer leads to a series of structural transitions on the surface.

**Figure 4:**
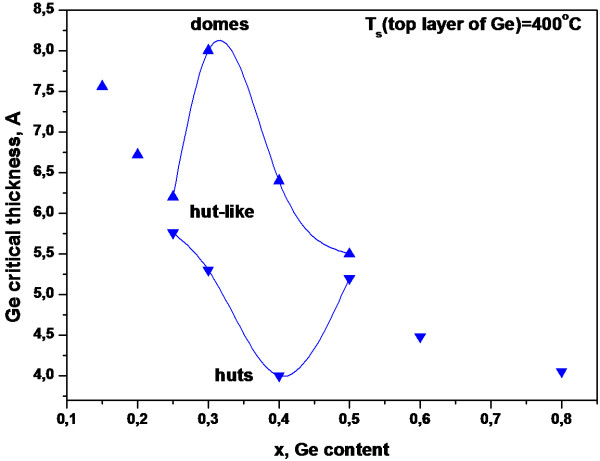
**The phase diagram of the growth for Ge/Ge**_***x***_**Si**_**1−*x***_**/Ge heterostructure.**

From the kinetic diagram describing the 2D to 3D transition of Ge_*x*_Si_1−*x*_ films, the values located below the 2D to 3D transition revealed the Ge_*x*_Si_1−*x*_ thickness region, such that Ge_*x*_Si_1−*x*_ layers were obtained the 2D dislocation-free pseudomorphic films. The presence of a thin strained Ge_*x*_Si_1−*x*_ film caused not only changes in critical thicknesses of the transitions, but also affected the properties of the germanium nanocluster array for the top Ge layer. Based on the RHEED data, the shape of the hut-like islands, which were not observed before between the hut and dome islands, was detected. Thus, shaped islands appear on the phase diagram in the range of *x* = 0.25 to *x* = 0.5 (Figure 4). Data were obtained on the growth of Ge/Ge_*x*_Si_1−*x*_/Ge heterostructures with the uniform array of islands (standard deviation is approximately 10%) in the second layer of the Ge film.

**Figure 5:**
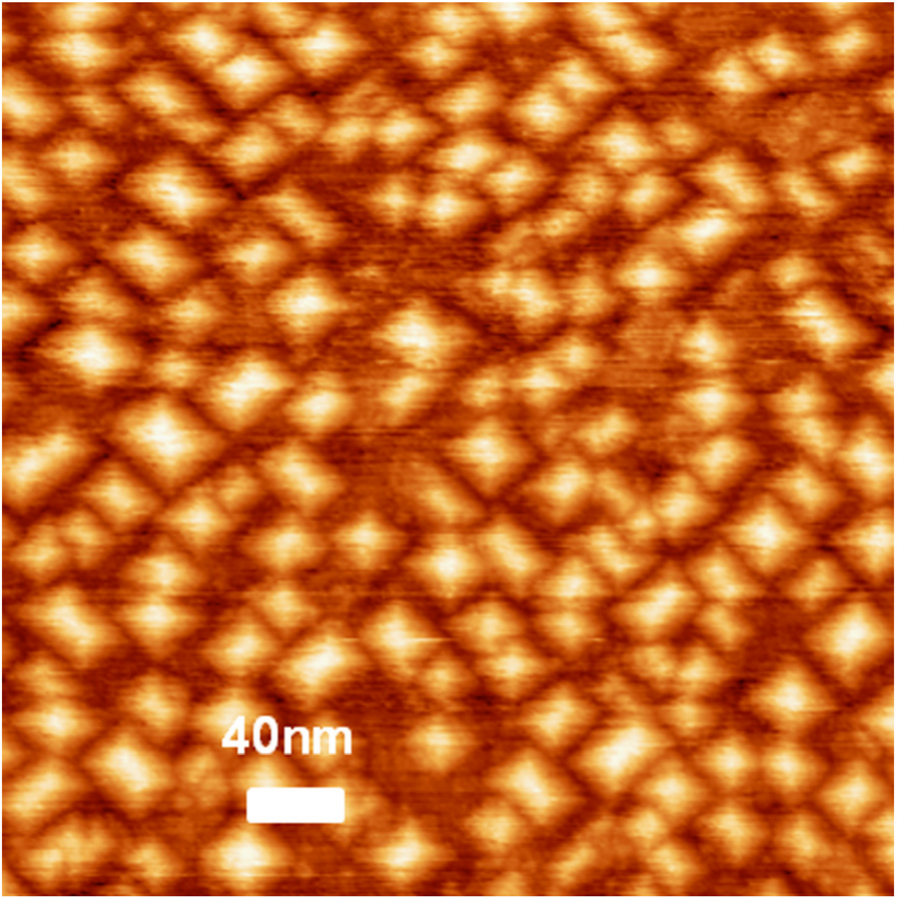
STM images of Ge islands.

The faceting by {105} planes changes upon deposition of several solid solution monolayers. Figure 5 shows an STM image of Ge islands (*d*_Ge_ = 0.3 nm) on Ge_0.3_Si_0.7_ (*d*_GeSi_ = 10 nm)/Ge hut-cluster layers (*d*_Ge_ = 0.6 nm) at 500°C. Germanium islands are formed as square-based pyramids on the solid solution surface similar to those reported in [20]. Their density is lower than the density of underlying initial *hut* clusters (9 × 10^10^ cm^−2^). Furthermore, bases of the pyramidal islands differ from those of the initial *hut* clusters; they are 20 nm in characteristic size. However, the size distribution of the pyramidal islands is much more uniform (approximately by a factor of 2) than that of the germanium *hut* clusters. The underlying germanium layer causes variations in not only parameters of the island array, but also in their faceting. The RHEED data indicate the presence of facets at a greater angle than plane {105}. While the base edges keep their orientations, these are, supposedly, planes {104} or {103}; their accurate identification needs further studies. The presence of intermediate hut-like shape of the islands is of interest both for structural and topological properties of the surface that affect the optical and electronic properties of the system as a whole.

**Figure 6:**
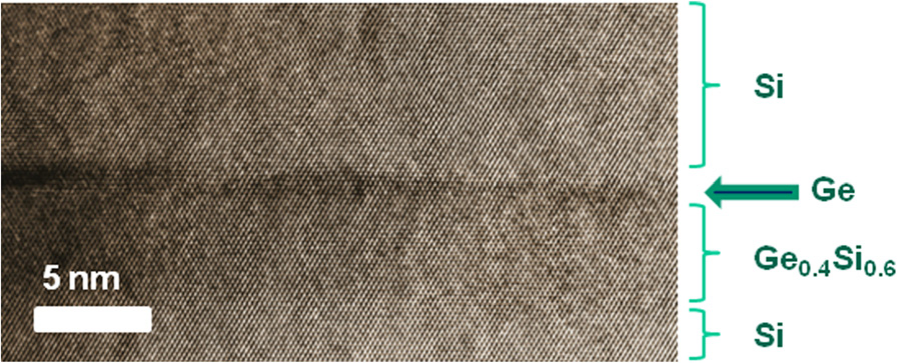
**High-resolution TEM image of Ge quantum dots (*d***_**Ge**_**= 0.3 nm) on 5-nm-thick Ge**_**0.4**_**Si**_**0.6**_** at 500°C.**

The crystal structure of Ge/Ge_*x*_Si_1−*x*_/Si heterolayers with quantum dots was analyzed using cross-sectional high-resolution transmission electron microscopy (TEM). Figure 6 shows a typical TEM image of such kind of heterostructures. Layers of germanium, solid solution, and silicon are rather different in contrast to allow them to be inspected individually. Neither of the layers contains any defects. Hence, the structure is elastically strained; plastic relaxation does not occur, and the germanium islands are of the characteristic *hut* cluster shape.

## Conclusions

The critical thickness of 2D to 3D transition was determined as a function of composition for different growth temperatures during the growth of solid solution Ge_*x*_Si_1−*x*_. Non-relaxed atomically smooth GeSi layers were used as an initial surface to fabricate Ge nanoislands. The results obtained make it possible to produce dislocation-free strained heterostructures Ge_*x*_Si_1−*x*_, where germanium quantum dots reside in quantum wells formed by layers of GeSi solid solution. It is shown that regular pyramidal germanium islands with different faceting and array properties are formed on the surface of the solid solution coverage over germanium *hut* clusters. The *n* of (2 × *n*) reconstruction was determined as a function of composition during the growth of solid solution Ge_*x*_Si_1−*x*_ in the Ge/Ge_*x*_Si_1−*x*_/Ge heterostructure. An increase in *n* from 8 to 14 was believed to take place at the enhancement of the Ge content in the Ge_*x*_Si_1−*x*_ film.

## Competing interests

The authors declare that they have no competing interests.

## Authors' contributions

AIN conceived the study, performed data analysis, and took part in discussions and interpretation of results; he also supervised and coordinated the research projects. VAT participated in the design of the study, carried out the experiments, performed data analysis, and took part in discussions and interpretation of results. SAT investigated the surface morphology by STM and took part in discussions and interpretation of results. AKG performed the HRTEM studies and took part in discussions and interpretation of results. OPP participated in the design of the study, took part in discussions and interpretation of results, and supervised the research project. All authors read and approved the final manuscript.
